# Stable Observation Generation of Retroreflective Landmarks for Utility-Tunnel Inspection Robots

**DOI:** 10.3390/s26196010

**Published:** 2026-09-23

**Authors:** Jicong Wang, Hongpeng Li, Jianhua Zhang, Siyuan Chen, Lihui Xu, Min Xie, Yue Long, Jiangpeng Shu

**Affiliations:** 1 School of Automation, Hangzhou Dianzi University, Hangzhou 310018, China; 2 School of Resource and Safety Engineering, Central South University, Changsha 410083, China; 3 Jiangxi Copper Group Co., Ltd., Guixi 335424, China; 4 Jiangxi Copper Group Yinshan Mining Co., Ltd., Dexing 334200, China; 5 College of Civil Engineering and Architecture, Zhejiang University, Hangzhou 310058, China

**Keywords:** retroreflective landmarks, utility tunnel, LiDAR perception, inspection robot, stable observation, temporal tracking, map association

## Abstract

**Highlights:**

**What are the main findings?**

- A scene-aware retroreflective-landmark pipeline that combines intensity and geometry filtering, sparse-candidate recovery, and temporal confirmation to suppress false positives while retaining stable observations in a real underground utility-tunnel setting.
- Annotated real LiDAR data validate observation quality, false-positive suppression, temporal stability, map-association availability, and runtime.

**What are the implications of the main findings?**

- Stable landmark observations provide a practical sensing interface for reflector-map association in repetitive, GNSS-denied corridors.
- The study evaluates the observation layer rather than absolute localization accuracy; external ground truth remains necessary for a localization claim.

**Abstract:**

Utility tunnels are narrow, repetitive, and GNSS-denied, while reflective infrastructure can create false landmark detections. This paper presents a lightweight observation method for low-cost retroreflective landmarks on inspection robots. The first contribution combines intensity filtering with local geometric verification to suppress reflective-background interference. The second combines sparse-candidate recovery with temporal tracking and state confirmation to retain stable observations under sparse returns and short occlusions. On annotated real LiDAR data, the method achieves 88.7% precision, an F1 score of 80.3%, and a mean processing time of 1.57 ms. The resulting observations are associated with a pre-built reflector map; their real-site contribution to absolute localization accuracy requires external trajectory ground truth and is not claimed here.

## 1. Introduction

Utility tunnels and underground cable galleries constitute critical urban infrastructure, housing power, communication, water, and gas systems. Inspection robots are increasingly deployed in such constrained environments to reduce manual inspection risk and improve maintenance coverage [1,2,3,4,5,6,7,8]. Unlike open industrial floors or outdoor roads, utility tunnels are long, narrow, repetitive, and mostly GNSS-denied. Cable racks, pipes, brackets, and walls are repeatedly arranged along the longitudinal direction, so different positions may produce similar LiDAR geometry.

LiDAR SLAM and LiDAR–inertial odometry have achieved strong performance in general robotic mapping. Representative systems such as LOAM, Cartographer, LeGO-LOAM, LIO-SAM, and FAST-LIO2 improve odometry and mapping through feature extraction, submap optimization, ground constraints, factor graphs, and direct LiDAR–inertial coupling [9,10,11,12,13,14,15]. However, long corridors, tunnels, and other structurally degraded environments remain challenging because geometric constraints along the corridor direction are weak. Recent work on adverse or degenerate environments has therefore explored degradation-aware weighting, tunnel-specific mapping, radar/LiDAR/inertial integration, and wheel-encoder assistance [16,17,18,19,20,21,22].

Artificial landmarks are an effective way to introduce additional observability. Retroreflective markers are low-cost, easy to deploy, and produce strong LiDAR intensity responses. Prior systems have used reflector markers, artificial landmarks, LiDAR fiducials, and pole landmarks for robust localization [23,24,25,26,27,28,29,30]. Nevertheless, utility-tunnel scenes contain many metallic racks, pipes, equipment shells, and other high-reflectance backgrounds. A simple intensity threshold may recover many candidates but also inject false landmarks into the back-end. In repetitive corridors, such false observations may be more harmful than missed detections because they can amplify map-matching ambiguity.

This paper therefore focuses on stable retroreflective-landmark observation generation rather than on a new navigation back end. The goal is to convert raw high-intensity point clusters into clean, temporally continuous landmark observations with low false-positive rates. Reflector-map association is treated as a downstream interface; absolute localization correction and trajectory evaluation are outside the scope of this study.

The main contributions are as follows:

1. We develop a scene-aware observation chain that combines intensity filtering, local geometric verification, sparse-candidate recovery, multi-frame tracking, and state confirmation to convert ambiguous high-reflectance returns into stable retroreflective-landmark observations in repetitive utility-tunnel scenes.
2. We validate the observation chain on annotated real LiDAR data through false-positive suppression, temporal stability, runtime, and reflector-map association availability; no real-site absolute-localization claim is made.

## 2. Related Work

### 2.1. LiDAR Localization in Degenerate Environments

LiDAR odometry estimates ego-motion by matching consecutive point clouds to each other or to a prior map. ICP and its variants remain fundamental registration tools [31,32], while incremental smoothing methods provide back-end optimization support [33]. In corridors and tunnels, however, scan matching may become under-constrained along the dominant axis. Recent tunnel and adverse-environment studies emphasize that additional constraints, robust weighting, or auxiliary sensors are needed in such cases [17,18,19,20,21,22].

### 2.2. Retroreflective and Artificial Landmark Localization

Retroreflective markers and artificial landmarks can provide reliable reference points when natural geometry is insufficient. Kurz and Scherer introduced reflector markers into LiDAR SLAM to support areas where geometry alone is inadequate [23]. Wang et al. proposed SORLA for lightweight LiDAR localization with artificial reflectors [24]. Zeng et al. combined ICP with artificial landmarks [25], and Xu et al. studied robust mobile-robot localization through LiDAR measurement and matching in ongoing scenes [34]. LiDARTag and intensity-image fiducial systems further show that LiDAR intensity can support fiducial detection and pose estimation [26,27].

### 2.3. LiDAR Intensity and Point-Cloud Target Detection

LiDAR intensity is related to surface reflectance but is also affected by range, incidence angle, sensor settings, and environmental conditions [35,36,37]. Recent work also indicates that feature quality strongly influences pose estimation error in LiDAR point-cloud matching [38]. Deep learning-based point-cloud segmentation and detection provide powerful alternatives [39,40,41], but they often require large annotated datasets and may be less convenient for low-cost deployment. This paper therefore uses lightweight intensity, geometry, and temporal tracking constraints tailored to utility-tunnel retroreflective markers.

### 2.4. Temporal Tracking and Stable Observations

Single-frame detection can flicker due to occlusion, sparse hits, or background interference. Multiple-object tracking literature highlights the importance of prediction, association, and track management [42,43]. Assignment and density clustering are also common tools for target association and extraction [44,45]. In retroreflective landmark localization, stable observations are more important than raw detections because unstable or false landmarks can degrade downstream localization.

## 3. Materials and Methods

### 3.1. Problem Definition

An inspection robot equipped with a LiDAR, an inertial measurement unit, and odometry runs in a long utility tunnel. The point cloud at time *t* is denoted as $P_{t}=\left\{p_{i}\right\}$, where each point is $p_{i}=\left(x_{i},y_{i},z_{i},I_{i}\right)$ and $I_{i}$ is the LiDAR return intensity. A pre-built reflector map is denoted as $M=\{m_{k}\}$, where $m_{k}\in R^{3}$ is the map-frame coordinate of a retroreflective marker. The objective is to extract stable observations $O_{t}=\left\{o_{j}\right\}$ and associate them with *M* so that the observations serve as localization constraints.

### 3.2. Sensor Setup and Coordinate Configuration

The real-data sequences were acquired with a Livox Mid-360 LiDAR (Livox Technology Company Limited, Shenzhen, China). The fixed LiDAR-to-body/IMU mounting transform used by the deployed ROS system has a translation of (0.187,0,0) m and roll–pitch–yaw angles of (0°, 18°, 0°). The supplied forward offset of 187 mm is reported here as 0.187 m. In the notation used below, this installed transform is represented by $\left(R_{LI},t_{LI}\right)$; its direction follows the deployed ROS transform convention. The transform was kept fixed for all reported real-data results. No distance-dependent intensity compensation was applied in the reported proposed-method evaluation; the intensity gate was applied directly to the recorded LiDAR return intensity. The deployed processing pipeline used ROS Noetic (ros_comm 1.17.4) and RViz 1.14.26; the bounded synthetic stress test used Gazebo 11.15.1; and the data-processing scripts used Python 3.8.10.

### 3.3. System Overview

The proposed pipeline contains three core observation stages: point-cloud preprocessing and high-intensity filtering, candidate generation, and candidate fusion with multi-frame tracking. Reflector-map association and localization-output bridging are optional downstream interfaces used to assess the usability of stable observations. Figure 1 summarizes the complete engineering pipeline.

### 3.4. High-Intensity Filtering and Candidate Generation

The high-intensity point set is obtained as

(1) $$P_{I}\left(t\right)=\{p_{i}\in P_{t}|I_{i}\geq \tau_{I},\;r_{\min}\leq ∥p_{i}{∥}_{2}\leq r_{\max},\;z_{\min}\leq z_{i}\leq z_{\max}\}.$$

Density clustering is then applied on the horizontal plane. For each cluster $C_{l}$, geometric statistics are computed as

(2) $$\Delta x_{l}=x_{l,\max}-x_{l,\min},\;\Delta y_{l}=y_{l,\max}-y_{l,\min},\;\Delta z_{l}=z_{l,\max}-z_{l,\min},$$

(3) $$\begin{array}{cccc} \rho_{l} & =\max(\Delta x_{l},\Delta y_{l}), & \sigma_{xy,l} & =\sqrt{\mathrm{var}\left(x_{l}\right)+\mathrm{var}\left(y_{l}\right)},\\ a_{l} & =\frac{\Delta z_{l}}{\max(\rho_{l},\epsilon )}, & I_{\mathrm{mean},l} & =\frac{1}{|C_{l}|}\sum_{p_{i}\in C_{l}}I_{i}. \end{array}$$

A dense candidate is accepted only when the point count, vertical span, horizontal scale, planar dispersion, aspect ratio, and mean intensity all satisfy the geometric gate:

(4) $$\begin{array}{cc} C_{l}\in C_{t}^{\mathrm{dense}}\Leftrightarrow & |C_{l}|\geq n_{c},\;\Delta z_{l}\geq h_{\min},\;\rho_{l}\leq \rho_{\max},\\ \; & \sigma_{xy,l}\leq \sigma_{\max},\;a_{\min}\leq a_{l}\leq a_{\max},\;I_{\mathrm{mean},l}\geq I_{\min}. \end{array}$$

Sparse candidates recover high-intensity but low-hit targets that would otherwise be missed by dense clustering. For an unclaimed sparse point set $S_{l}$, the relaxed gate is

(5) $$S_{l}\in C_{t}^{\mathrm{sparse}}\Leftrightarrow \left|S_{l}\right|\geq n_{s},\;\rho_{l}\leq \rho_{s},\;\Delta z_{l}\geq h_{s},\;\sigma_{xy,l}\leq \sigma_{s},\;I_{\mathrm{mean},l}\geq I_{s}.$$

For near-range sparse recovery, an additional local-background contrast check is used when background statistics are available:

(6) $$B_{l}=\{p_{i}\in P_{t}|\rho_{l}<∥p_{i,xy}-c_{l,xy}{∥}_{2}\leq \rho_{\mathrm{bg}},\;|z_{i}-z_{l,c}\left|\leq h_{\mathrm{bg}},\;p_{i}\notin S_{l}\right\},$$

(7) $$I_{\mathrm{bg},l}=\mathrm{median}\left(\left\{I_{i}|p_{i}\in B_{l}\right\}\right),\;\frac{I_{\mathrm{mean},l}}{\max(I_{\mathrm{bg},l},\epsilon_{I})}\geq \gamma .$$

When $B_{l}$ is empty, the contrast term is not applied as a hard gate; the sparse path instead relies solely on intensity, scale, and vertical-geometry constraints. The system fuses dense and sparse candidates within $\epsilon_{f}$ by point-count-weighted center averaging. Table 1 lists the proposed observation-module parameters used for real-data observation generation and downstream map association.

### 3.5. Temporal Tracking and State Confirmation

Candidates from dense and sparse paths are fused to avoid duplicate outputs. A prediction–association–update loop maintains landmark tracks. Each track uses a constant-velocity state

(8) $$x_{j,t}={\left[p_{x},p_{y},p_{z},v_{x},v_{y},v_{z}\right]}^{T},\;x_{j,t}^{-}=F\left(\Delta t\right)x_{j,t-1},\;F\left(\Delta t\right)=\left[\begin{array}{cc} I_{3} & \Delta tI_{3}\\ 0 & I_{3} \end{array}\right].$$

For a candidate center $c_{l,t}$, the track-association cost is

(9) $$d_{jl}={∥c_{l,t}-Hx_{j,t}^{-}∥}_{2},\;H=\left[I_{3}\;\;0\right].$$

The global one-to-one assignment is solved on the cost matrix $D=[d_{jl}]$, and matches outside the distance gate are rejected. For an accepted match, the candidate center is treated as a position observation:

(10) $$\begin{array}{cc} z_{l,t} & =Hx_{j,t}+v_{l,t},\;v_{l,t}∼N\left(0,R_{c}\right), \end{array}$$

(11) $$\begin{array}{cc} K_{j,t} & =P_{j,t}^{-}H^{T}{\left(HP_{j,t}^{-}H^{T}+R_{c}\right)}^{-1}, \end{array}$$

(12) $$\begin{array}{cc} x_{j,t} & =x_{j,t}^{-}+K_{j,t}\left(z_{l,t}-Hx_{j,t}^{-}\right), \end{array}$$

(13) $$\begin{array}{cc} P_{j,t} & =\left(I-K_{j,t}H\right)P_{j,t}^{-}. \end{array}$$

The raw confidence combines intensity, geometry, temporal continuity, and prediction reliability:

(14) $$q_{j,t}=0.35s_{I}+0.25s_{G}+0.20\frac{\min(n_{j},5)}{5}+0.20s_{P}.$$

To suppress single-frame fluctuations, the system smooths track confidence with an exponential moving average:

(15) $$Q_{j,t}=0.4q_{j,t}+0.6Q_{j,t-1}.$$

For unmatched tracks, the confidence decays as $Q_{j,t}=0.92Q_{j,t-1}$. The track manager classifies tracks into tentative, stable, degraded, and lost states according to observation continuity and confidence, and publishes only stable or sufficiently reliable degraded observations to the localization side. Figure 2 summarizes the state-transition logic.

### 3.6. Reflector-Map Association Interface

For downstream use, each stable observation in the LiDAR/body frame can be associated with a pre-built reflector map using an externally supplied pose prediction. For a map landmark $m_{i}^{W}$, a LiDAR observation centroid $z_{i}^{L}$, pose prediction $T_{t}^{-}$, and LiDAR–IMU extrinsic $\left(R_{LI},t_{LI}\right)$, the observation relationship is

(16) $$z_{i}^{L}=R_{LI}^{T}\left(R_{WI}^{T}\left(m_{i}^{W}-p_{WI}\right)-t_{LI}\right)+v_{i}.$$

The predicted map-frame observation is associated by nearest-neighbor gating:

(17) $${\hat{m}}_{j,t}=T_{t}^{-}z_{j}^{L},\;k^{*}=\arg\min_{k}∥{\hat{m}}_{j,t}-m_{k}{∥}_{xy},\;{∥{\hat{m}}_{j,t}-m_{k^{*}}∥}_{xy}<d_{m}.$$

This interface retains the landmark identity, centroid, confidence, and association residual for a downstream localization system. It does not itself estimate a robot trajectory or claim a single-landmark pose solution. The real-data results therefore report observation accuracy and association availability rather than absolute localization error.

### 3.7. Use of Generative AI

During the preparation of this manuscript, the authors used OpenAI Codex (GPT-5) (OpenAI, San Francisco, CA, USA; accessed July–August 2026) for language polishing, manuscript organization, LaTeX formatting, and consistency checking of figures, tables, and code descriptions. The authors reviewed and edited all AI-assisted outputs and take full responsibility for the content of the manuscript. No generative AI tool was used to generate experimental data, conduct experiments, or replace the authors’ interpretation of the results.

## 4. Results

### 4.1. Datasets and Evaluation Protocol

The evaluation uses real LiDAR observation data, engineering-chain checks, and supplementary sequences acquired in a real underground utility-tunnel setting. The main real sequence lasts 89.99 s and contains 846 exported observations and 18,016 IMU records. Manual annotation covers 60 frames, including 42 positive frames, 18 negative frames, 75 visible target instances, and 43 reflector IDs. Because the real bag has no external trajectory ground truth, the evaluation focuses on observation accuracy, false-positive suppression, temporal continuity, and map-association availability rather than absolute localization error. Figure 3 shows the real scene and point-cloud view.

### 4.2. Real-Data Observation Accuracy, Baselines, and Distance Behavior

Table 2 reports observation accuracy under XY and 3D matching criteria. XY matching is emphasized because the robot mainly navigates on a horizontal plane.

Figure 4 shows a representative annotated frame.

For a fair primary comparison, every method in Table 3 uses the same candidate intensity threshold of 140, the same 0.08 m clustering radius, and the same 0.15 m XY matching gate. The threshold was fixed before metric aggregation and was not separately optimized for individual methods. The SORLA-style comparator implements only high-reflectance extraction and cluster compactness filtering, rather than the original method’s complete localization system. The proposed method reduces false positives while retaining a usable observation rate.

Figure 5 shows the threshold-sweep precision–recall behavior of the single-frame baselines. It is a sensitivity visualization; the fixed-threshold comparison used for the primary claim is Table 3.

The controlled module variants in Table 4 clarify the role of the individual components. Relative to dense-only geometric candidates, sparse recovery restores 10 ground-truth instances at the candidate stage: four at 0.9–2 m and six at 2–4 m. Relative to the single-frame geometric variant, temporal confirmation reduces FPs from 72 to 7, while reducing TPs from 74 to 55. This intentional precision–recall trade-off is important in repetitive corridors, where false reflector observations can create ambiguous map associations. The dense-only and sparse-enabled variants are paired to evaluate candidate recovery, whereas the single-frame and full variants are paired to evaluate temporal confirmation; they are not presented as a strictly cumulative sequence.

Table 5 shows that recall is 82.1% at 2–4 m and 66.7% at 0.9–2 m. The few samples beyond 4 m are reported only as supplementary observations.

### 4.3. Supplementary 130-Frame Real-Sequence Evaluation

To assess whether the primary result is retained on a separately recorded sequence, 130 manually reviewed frames were evaluated under the same protocol. This supplementary recording contains 46 positive frames with 47 visible reflector instances and 84 negative frames. All compared methods use the same candidate intensity threshold of 140, clustering radius of 0.08 m, and XY matching gate of 0.15 m; the threshold was fixed before the rerun. Thus, the comparison does not use a more permissive intensity setting for either the proposed method or a baseline.

Table 6 reports the rerun results. The proposed observation chain produces 33 TPs, no false candidates in positive frames, and 14 FNs, corresponding to a precision of 1.000, a recall of 0.702, and an F1 of 0.825. Its 95% Wilson interval for precision is [0.896, 1.000] and for recall it is [0.560, 0.813]. Compared with fixed-threshold clustering and the SORLA-style high-reflectance cluster baseline, it markedly reduces the negative-frame false-alarm rate, from 46.4% to 1.2%. The single-frame geometry variant retains higher recall but produces a 13.1% negative-frame false-alarm rate. These results support the intended operating point of the proposed observation chain: conservative output with low false-positive risk, rather than maximum single-frame recall.

### 4.4. Synthetic High-Return Clutter Stress Test

To provide a bounded supplementary check for the reflective-background concern, three no-landmark Gazebo scenes were constructed from the tunnel test world: a high-return planar panel, a horizontal pipe-like high-return object, and a high-return floor patch. The proposed observation module processed 100, 99, and 101 scans, respectively. Since no true reflector landmark was placed in these scenes, every output would be a false positive. As summarized in Table 7, no observation or stable output was produced in the three prescribed high-return conditions.

This simulation is a stress test of the implemented intensity, scale, vertical-geometry, and temporal-confirmation gates. It does not calibrate material bidirectional reflectance, wet-surface multipath, mirror reflections, or sensor saturation; it therefore cannot establish generalization to every metallic, wet, or specular field background. Moreover, illumination was not varied because LiDAR intensity is an active sensing measurement. The real-sequence results remain the primary evidence for utility-tunnel operation.

### 4.5. Downstream Map-Association Availability

The observation outputs are also evaluated for downstream map-association availability. Of the 42 positive frames, 36 (85.7%) contain at least one true matched reflector observation. Twenty-five frames (59.5%) contain at least two annotated visible reflectors. In 15 of these 25 co-visible frames (60.0%), at least two reflector identities are correctly matched; in 12 frames (48.0%), all visible reflector identities are correctly matched. These are observation-and-association availability measures, not a multi-landmark geometric-localization experiment. Table 8 provides the corresponding accounting.

To quantify the operational consequence of temporal confirmation, the same 0.15 m XY gate was applied to the recorded outputs of the controlled variants. Table 9 shows that the single-frame geometric variant provides at least one correct association in all 42 positive frames and at least two correct associations in 24 of 25 co-visible frames. The proposed observation chain retains at least one correct association in 36 of 42 positive frames and at least two correct associations in 15 of 25 co-visible frames. In exchange, it reduces positive-frame false candidates from 72 to 7 and negative-frame false-alarm frames from 11 of 18 to 1 of 18. Thus, temporal confirmation deliberately trades association availability for a substantially cleaner landmark stream. Because no external trajectory ground truth is available, this analysis does not measure the effect of the recall reduction on absolute robot-localization performance.

### 4.6. Temporal Stability, False Positives, and Runtime

The full output sequence contains 846 observations, of which 831 (98.2%) are stable. The mean, median, and maximum track lengths are 13.0, 11, and 41 frames, respectively. Figure 6 visualizes reflector-ID continuity, showing that stable identities are maintained across consecutive frames.

In the 18 negative frames without visible reflectors, only one frame contains a false positive, giving a 5.56% false-alarm rate. Figure 7 shows the spatial distribution of TP, FP, and FN cases in the LiDAR frame.

Runtime is evaluated from 407 timing records. The mean, median, 95th percentile, and maximum processing times are 1.57, 1.50, 2.30, and 3.10 ms, respectively, with no spike above 50 ms.

## 5. Discussion

The main result is an improvement in the quality of retroreflective-landmark observations. Compared with single-frame high-reflectance extraction, the proposed output is more conservative: it trades some recall for substantially fewer false positives. This design choice is motivated by the risk that false landmarks can amplify association ambiguity in repetitive corridors.

The present paper does not claim a controlled real-world localization improvement because the real bag lacks external trajectory ground truth and a synchronized reflector-disabled output. The map-association interface is included to define a practical downstream use of the stable observations, but it is not evaluated as a complete localization back end. Tunnel and long-corridor studies nevertheless motivate low-false-positive artificial-landmark observations because repetitive, feature-poor geometry can weaken conventional LiDAR/LIO constraints [17,18,19,20,21,22].

The limitations are clear. First, although the 130-frame supplement expands the manual review, all real sequences were acquired in the same real underground utility-tunnel setting and the real annotated dataset remains limited, especially for long-range targets. Second, the five-scan supplementary protocol is not a continuous long-track evaluation. Third, the synthetic high-return stress test does not establish generalization to every metallic, wet, or specular background because it does not model calibrated material reflectance or wet-surface multipath. Finally, the current real bag lacks external trajectory ground truth. Future work should pursue three directions: (1) expanded annotation across multiple tunnel scenes and reflective-background conditions; (2) external ground truth from motion capture, total stations, or high-grade SLAM systems; and (3) controlled reflector-enabled/reflector-disabled localization experiments with repeated runs.

## 6. Conclusions

The main conclusions are as follows:

1. **Stable observation generation.** The proposed scene-aware observation chain combines intensity filtering, local geometric verification, sparse-candidate recovery, temporal tracking, and state confirmation. On annotated real LiDAR data, it achieves 88.7% precision, 73.3% recall, and an 80.3% F1 score under a 0.15 m XY threshold. The stable-output ratio reaches 98.2%, 85.7% of positive frames contain at least one true reflector observation, and the mean processing time is 1.57 ms. On the separately recorded 130-frame sequence under the same threshold, the proposed observation chain yields no false candidates in positive frames and a 1.2% negative-frame false-alarm rate; this additional evidence remains limited to the same real operating environment.
2. **Multi-reflector association availability.** Among 25 frames with at least two annotated visible reflectors, 15 frames contain at least two correctly matched identities and 12 match all visible identities. These values show that the stable outputs can support a reflector-map association interface; they do not constitute a multi-landmark or absolute-localization evaluation.
3. **Engineering significance and scope.** The method provides a lightweight observation layer for reflector-map association in repetitive, GNSS-denied tunnel-like environments. Absolute localization improvement remains to be evaluated with external ground truth and a controlled reflector-enabled/reflector-disabled protocol.

## Figures and Tables

**Figure 1:**
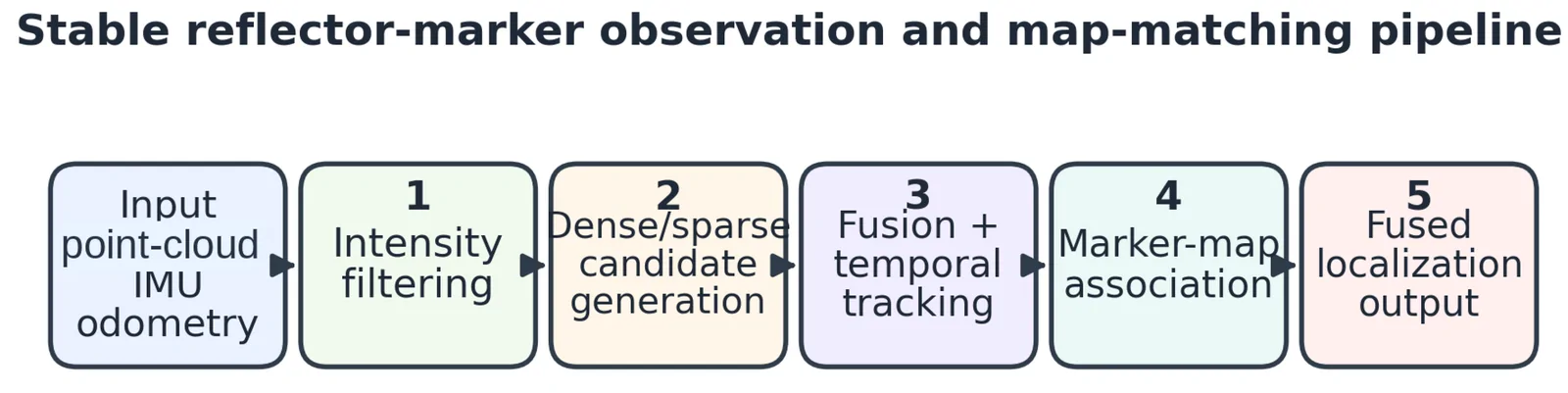
Engineering pipeline centered on retroreflective-landmark stable observation generation; map association and localization output are downstream interfaces.

**Figure 2:**
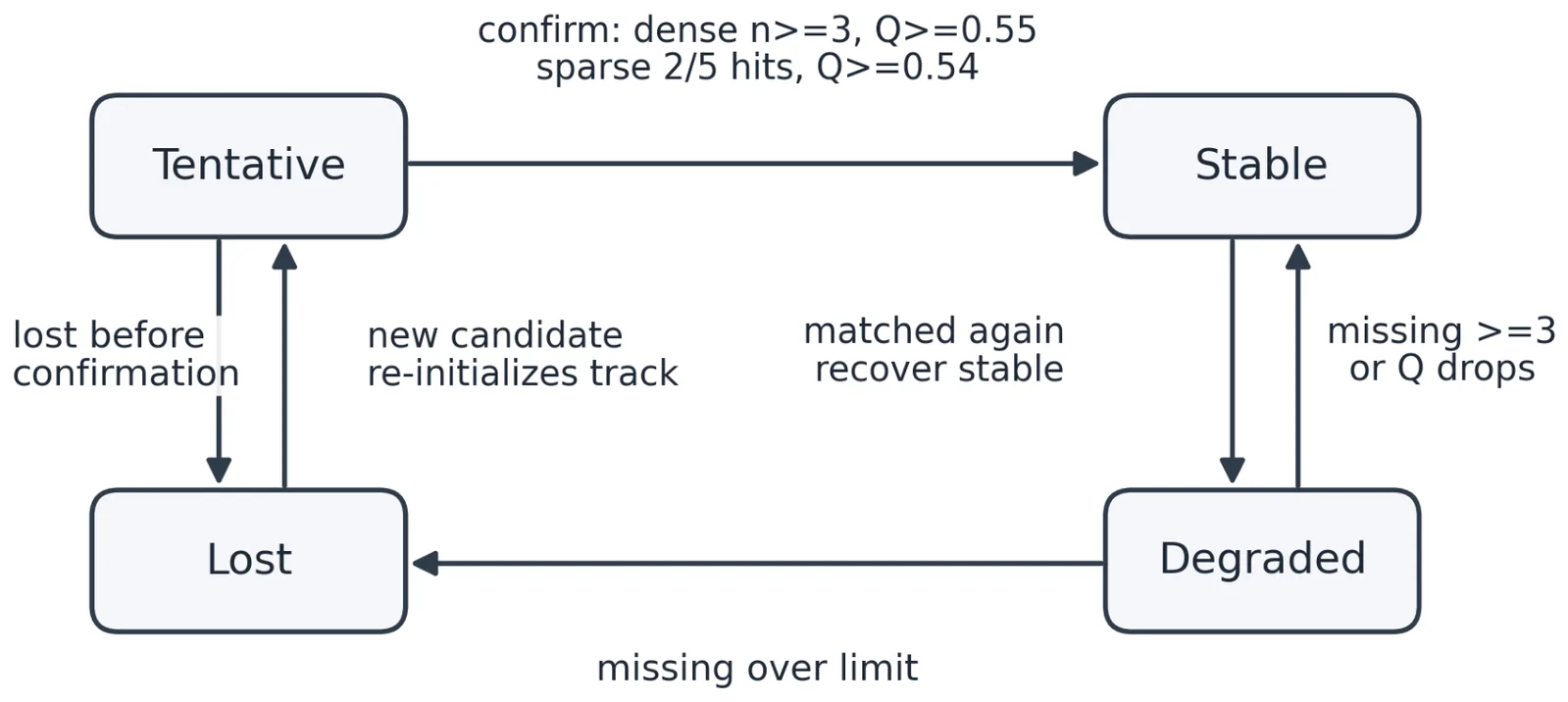
State-transition logic of the temporal track manager. Candidate tracks are promoted only after sufficient observations and confidence, degraded after short-term missing detections, and removed after persistent loss.

**Figure 3:**
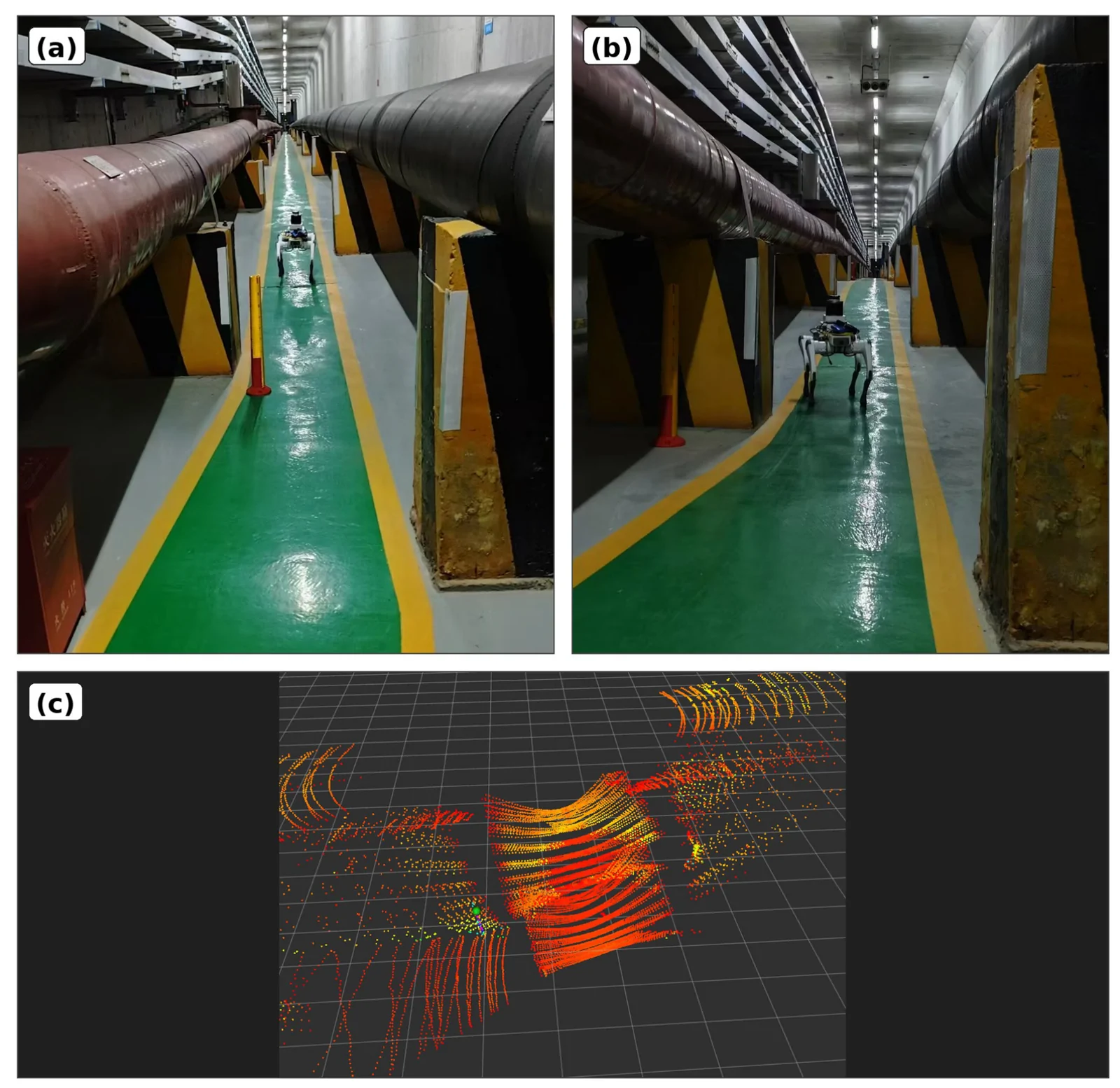
(**a**,**b**) Real underground utility-tunnel scene, illustrating the narrow corridor, repetitive geometry, pipe infrastructure, and reflector deployment considered in this study; (**c**) corresponding RViz point-cloud view. Colours in (**c**) are used for RViz visual rendering only and do not encode a detection class or quantitative metric.

**Figure 4:**
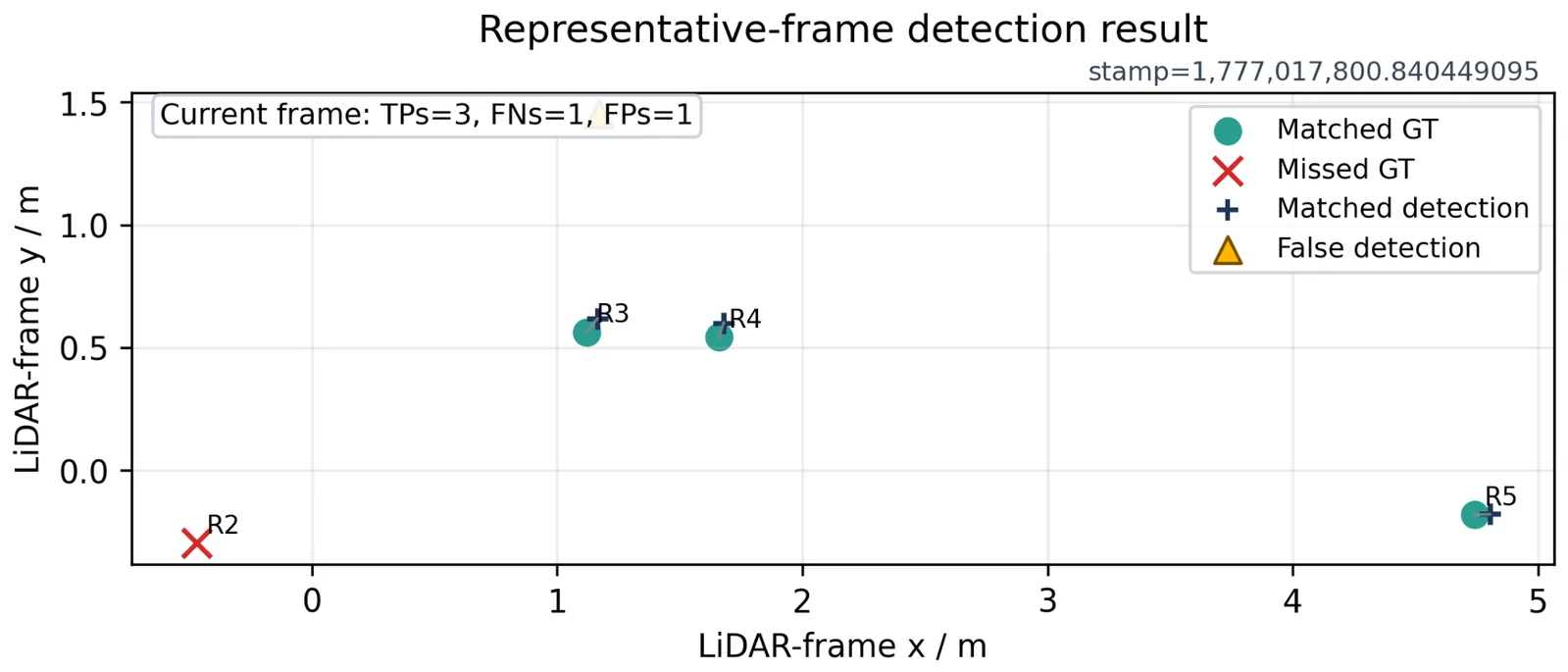
Detection visualization on annotated LiDAR frames, with true positives, false negatives, and false positives marked.

**Figure 5:**
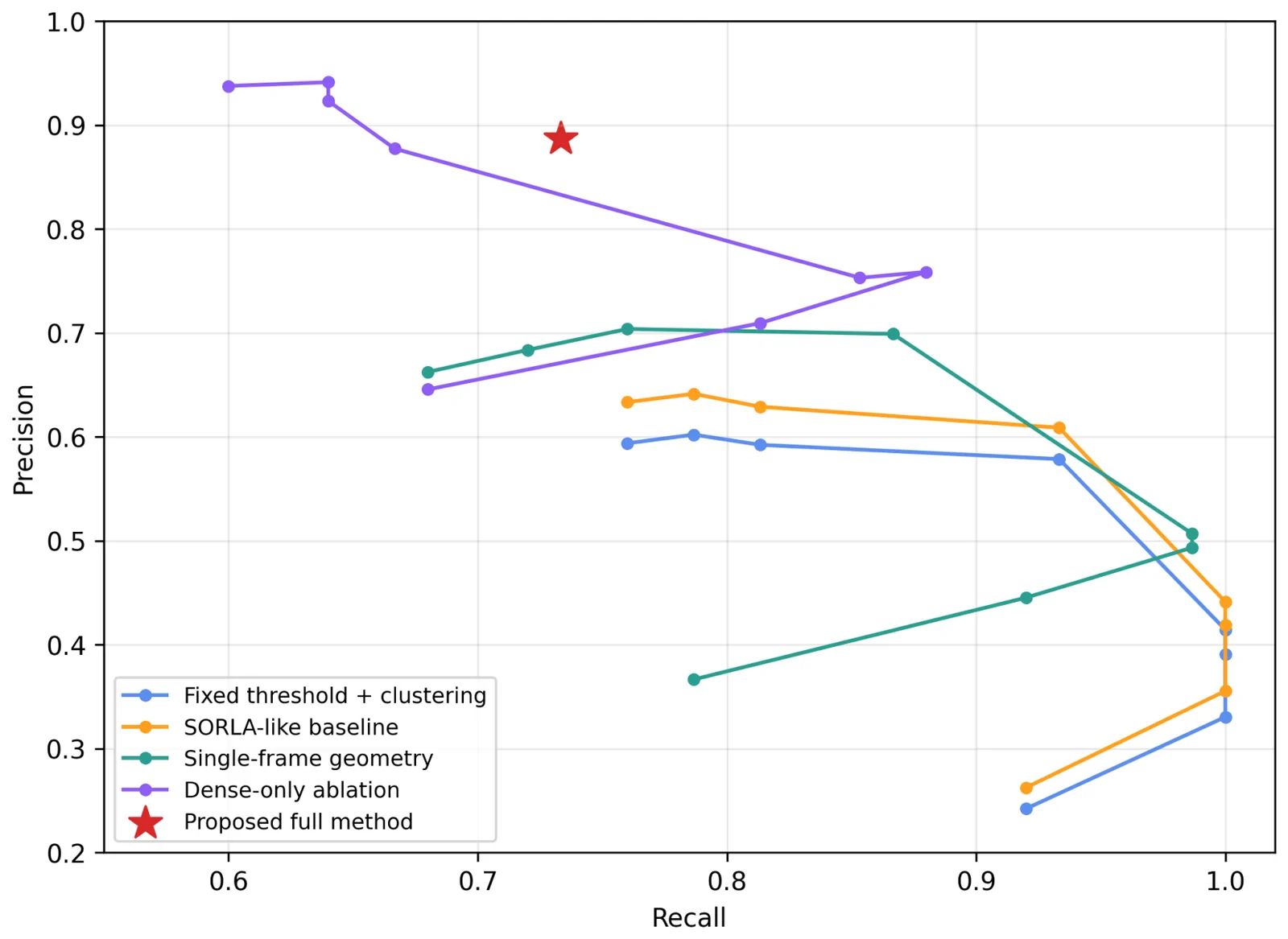
Precision–recall comparison between the proposed method and baseline high-reflectance extraction strategies.

**Figure 6:**
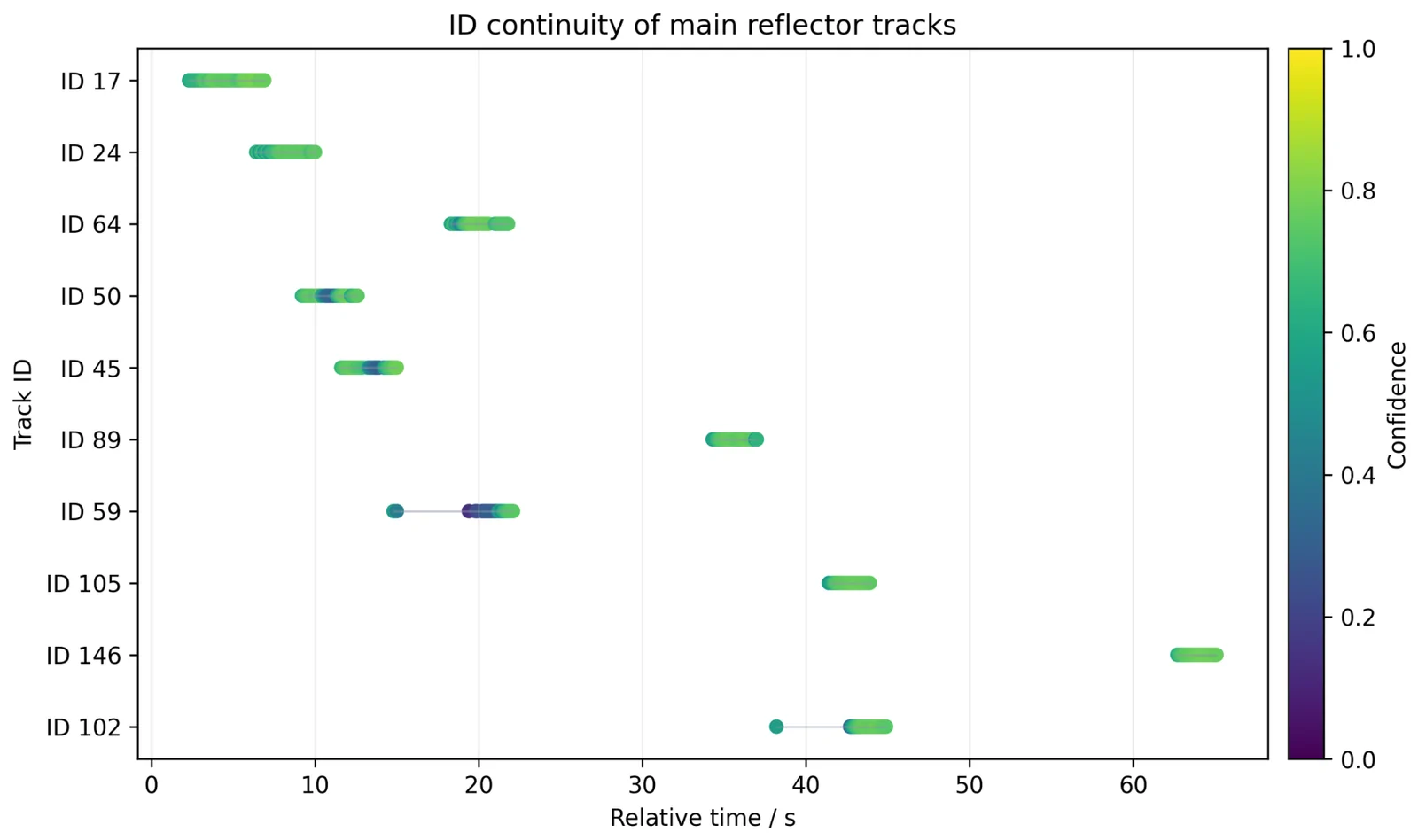
Temporal continuity of reflector track IDs after candidate fusion and state confirmation.

**Figure 7:**
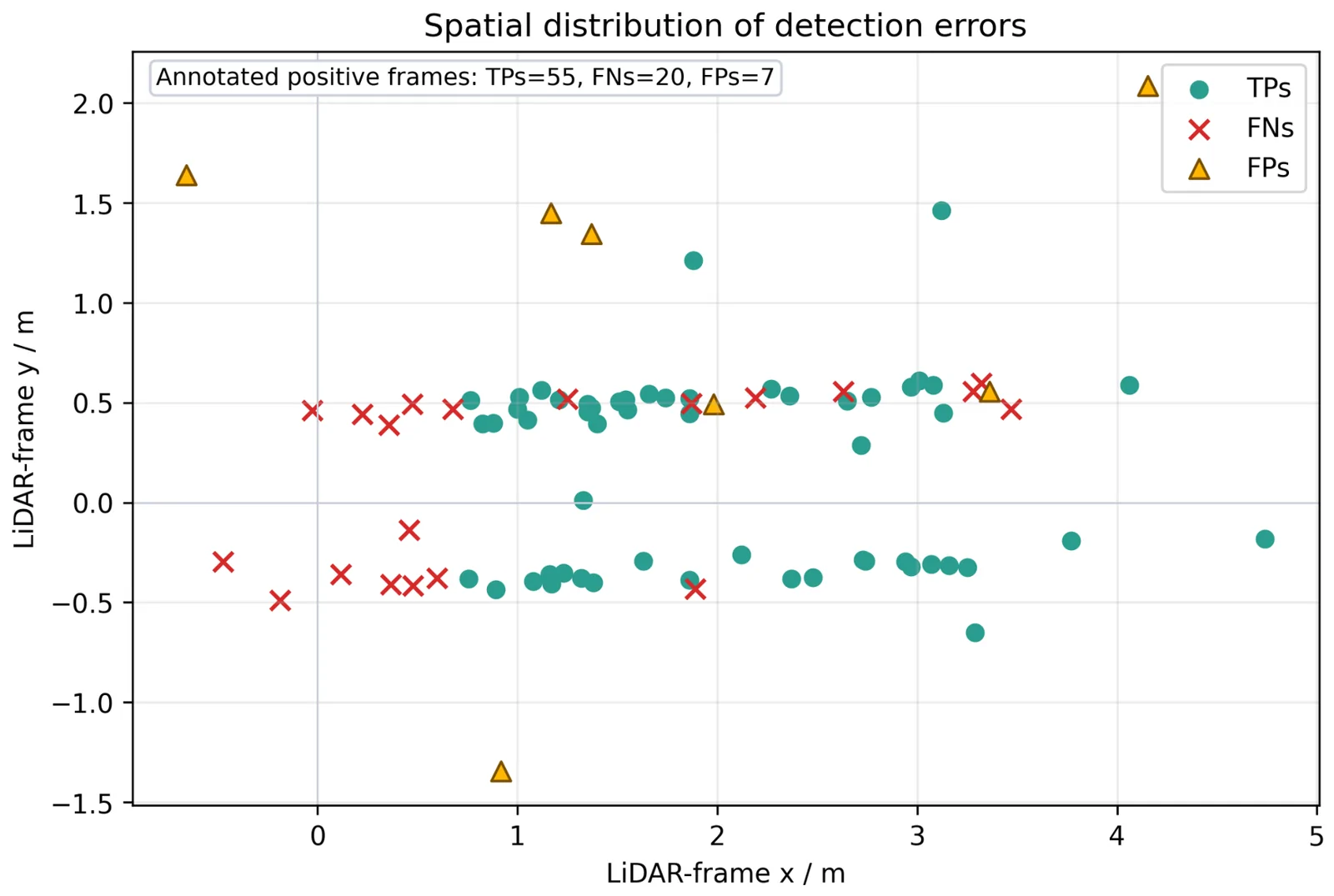
Spatial distribution of true positives (TPs), false positives (FPs), and false negatives (FNs) in the LiDAR coordinate frame.

**Table 1:** Parameters of the proposed observation module and downstream map association.

Category	Parameter	Value
Intensity filtering	Main intensity threshold $\tau_{I}$	140
Dense clustering	XY cluster radius $\epsilon_{c}$	0.08 m
Dense clustering	Minimum cluster size $n_{c}$	8 points
Dense geometry	Minimum height span $h_{\min}$	0.12 m
Dense geometry	Maximum horizontal scale $\rho_{\max}$	0.26 m
Dense geometry	Maximum planar dispersion $\sigma_{\max}$	0.06 m
Sparse recovery	Sparse XY aggregation radius $\rho_{s}$	0.09 m
Sparse recovery	Sparse recovery start range	2.0 m
Sparse recovery	Minimum sparse points $n_{s}$	2 points
Near-sparse recovery	Near-range interval	0.9–3.0 m
Near-sparse recovery	Background contrast ratio γ	1.10
Candidate fusion	Fusion radius $\epsilon_{f}$	0.12 m
Tracking	Maximum association distance $d_{\max}$	0.35 m
Tracking	Candidate-center measurement noise $R_{c}$	$0.10I_{3}$
Tracking	Confirmation condition	Dense: $n_{j}\geq 3$, $Q_{j,t}\geq 0.55$; sparse: 2 hits/5 frames, $Q_{j,t}\geq 0.54$
Tracking	Confidence EMA/missing decay	0.4/0.6; 0.92
Map association	Association radius $d_{m}$	2.0 m

**Table 2:** Observation accuracy under XY and 3D matching criteria.

Matching Criterion	Threshold	TP	FP	FN	Precision	Recall
XY plane	0.15 m	55	7	20	0.887	0.733
3D space	0.35 m	54	8	21	0.871	0.720

**Table 3:** Uniform-threshold comparison with high-reflectance extraction baselines under the XY matching criterion. All methods use an intensity threshold of 140, a clustering radius of 0.08 m, and a 0.15 m XY matching gate.

Method	$I_{\min}$	TP	FP	FN	Prec.	Rec.	F1
Proposed observation chain	140	55	7	20	0.887	0.733	0.803
Fixed intensity + clustering	140	75	106	0	0.414	1.000	0.586
SORLA-style reflective-cluster baseline	140	75	95	0	0.441	1.000	0.612
Single-frame geometric candidates	140	74	72	1	0.507	0.987	0.670

**Table 4:** Controlled module variants under the common threshold protocol. Sparse recovery is evaluated by comparing the dense-only and sparse-enabled candidate variants; temporal confirmation is evaluated by comparing the single-frame geometric and proposed variants.

Variant	TP	FP	FN	Precision	Recall	F1
Fixed intensity + clustering	75	106	0	0.414	1.000	0.586
Single-frame geometry + sparse recovery	74	72	1	0.507	0.987	0.670
Dense-only geometry (without sparse recovery)	64	21	11	0.753	0.853	0.800
Proposed observation chain	55	7	20	0.887	0.733	0.803

**Table 5:** Recall in different distance ranges.

Distance Range	TP	FN	Recall
0.9–2 m	30	15	0.667
2–4 m	23	5	0.821
>4 m	2	0	1.000

**Table 6:** Supplementary 130-frame real-sequence evaluation under the common intensity threshold of 140. FP+ denotes false candidates in positive frames; FP- denotes false-positive frames among 84 negative frames. The result is an observation-level comparison, not an absolute localization evaluation.

Method	TP	FP+	FN	Prec.	Rec.	F1	FP- Rate
Proposed observation chain	33	0	14	1.000	0.702	0.825	1.2%
Fixed intensity + clustering	46	17	1	0.730	0.979	0.836	46.4%
SORLA-style reflective-cluster baseline	46	17	1	0.730	0.979	0.836	46.4%
Single-frame geometry	43	5	4	0.896	0.915	0.905	13.1%

**Table 7:** Synthetic high-return clutter stress test in no-landmark Gazebo scenes. Every detector output would be a false positive. The tests are bounded module-level stress checks, not a substitute for multi-site real-scene validation.

Synthetic Clutter Condition	Processed Scans	False-Positive Frames	Mean FP/Scan	Stable-Output Frames	Stable-Output Rate
High-return planar panel	100	0 (0.00%)	0.00	0	0.00%
Horizontal pipe-like object	99	0 (0.00%)	0.00	0	0.00%
High-return floor patch	101	0 (0.00%)	0.00	0	0.00%

**Table 8:** Multi-reflector observation and association availability in annotated real-data frames. These metrics do not represent multi-landmark localization accuracy.

Metric	Value	Meaning
Annotated frames	60	Observation and proxy-constraint evaluation
Positive frames	42	Frames with visible reflector landmarks
Frames with at least one true matched reflector observation	36 (85.7%)	At least one association is available
Frames with at least two annotated visible reflectors	25 (59.5%)	Co-visible multi-reflector test subset
Co-visible frames with at least two matched identities	15/25 (60.0%)	At least two correct associations in the subset
Co-visible frames with all visible identities matched	12/25 (48.0%)	Complete observation association in the subset

**Table 9:** Frame-level association availability and false-candidate trade-off under the common 0.15 m XY gate. FP+ is the number of false candidates in positive frames; FP− is the number of false-positive frames among 18 negative frames. The table evaluates observation and association availability, not localization accuracy.

Variant	≥1 True/42	≥2 True/25	FP+/FP−
Single-frame geometry + sparse recovery	42/42 (100.0%)	24/25 (96.0%)	72/11/18
Dense-only geometry	40/42 (95.2%)	19/25 (76.0%)	21/3/18
Proposed observation chain	36/42 (85.7%)	15/25 (60.0%)	7/1/18

## Data Availability

Processed evaluation data, manual annotations, detector outputs, baseline metrics, timing records, and the proposed observation-chain implementation snapshot are included in the Supplementary Materials. The two raw LiDAR bags (3.72 GB) are not embedded because of file-size limitations; their identifiers and SHA-256 checksums are recorded in the accompanying data manifest. The raw data supporting the reported observation-level findings are available from the corresponding author upon reasonable request.

## Supplementary Materials

The following supporting information can be downloaded at: https://www.mdpi.com/article/10.3390/s26196010/s1. Supplementary Material S1: data-provenance manifest; aggregate CSV tables for all reported observation metrics; manual annotations; detector outputs; matching details; timing records; baseline grids; proposed observation-chain implementation snapshot; map-association interface; ROS launch and configuration files; evaluation scripts; and checksum/data-access manifests.

## Abbreviations

AGV	Automated guided vehicle
GNSS	Global navigation satellite system
ICP	Iterative closest point
IMU	Inertial measurement unit
LiDAR	Light detection and ranging
SLAM	Simultaneous localization and mapping

## References

[1] Jia Z., Liu H., Zheng H., Fan S., Liu Z. (2022). An Intelligent Inspection Robot for Underground Cable Trenches Based on Adaptive 2D-SLAM. Machines.

[2] Jia Z., Tian Y., Liu Z., Fan S. (2022). Condition Assessment of the Cable Trench Based on an Intelligent Inspection Robot. Front. Energy Res..

[3] Ma Z., Xiao J., Tang Y. (2021). Interactive Control of Power Inspection Robots Based on Laser SLAM Technology. Mach. Electron..

[4] Li H., Feng C., Xiao J., Zheng H., Jia Z. (2024). Tightly Coupled SLAM System for Intelligent Inspection Robots in Underground Cable Trenches. Comput. Meas. Control.

[5] Halder S., Afsari K. (2023). Robots in Inspection and Monitoring of Buildings and Infrastructure: A Systematic Review. Appl. Sci..

[6] Sjölander A., Belloni V., Ansell A., Nordström E. (2023). Towards Automated Inspections of Tunnels: A Review of Optical Inspections and Autonomous Assessment of Concrete Tunnel Linings. Sensors.

[7] Oguntoye K.S., Laflamme S., Sturgill R., Eisenmann D.J. (2023). Review of Artificial Intelligence Applications for Virtual Sensing of Underground Utilities. Sensors.

[8] Liu K.Q., Zhong S.S., Zhao K., Song Y. (2023). Motion Control and Positioning System of Multi-Sensor Tunnel Defect Inspection Robot: From Methodology to Application. Sci. Rep..

[9] Zhang J., Singh S. (2014). LOAM: Lidar Odometry and Mapping in Real-Time. Robotics: Science and Systems.

[10] Hess W., Kohler D., Rapp H., Andor D. Real-Time Loop Closure in 2D LIDAR SLAM. Proceedings of the IEEE International Conference on Robotics and Automation.

[11] Shan T., Englot B. LeGO-LOAM: Lightweight and Ground-Optimized Lidar Odometry and Mapping on Variable Terrain. Proceedings of the IEEE/RSJ International Conference on Intelligent Robots and Systems.

[12] Shan T., Englot B., Meyers D., Wang W., Ratti C., Rus D. LIO-SAM: Tightly-Coupled Lidar Inertial Odometry via Smoothing and Mapping. Proceedings of the IEEE/RSJ International Conference on Intelligent Robots and Systems.

[13] Xu W., Cai Y., He D., Lin J., Zhang F. (2022). FAST-LIO2: Fast Direct LiDAR-Inertial Odometry. IEEE Trans. Robot..

[14] Xue G., Li R., Zhang Z., Liu R. (2023). Research Status and Development Trend of SLAM Algorithms Based on 3D LiDAR. Inf. Control.

[15] Yue X., Zhang Y., He M. (2023). LiDAR-Based SLAM for Robotic Mapping: State of the Art and New Frontiers. arXiv.

[16] Li Z., Wu X., Cui J., Xu Y., Liu R., Tu R., Jiang W. (2026). Tunnel-SLAM: Low-Cost LiDAR/Vision/RTK/Inertial Integration on Vehicles for Roadway Tunnels. Electronics.

[17] Chen Z., Chen H., Qi Y., Zhong S., Feng D., Jin W., Wen W., Liu M. RELEAD: Resilient Localization with Enhanced LiDAR Odometry in Adverse Environments. Proceedings of the IEEE International Conference on Robotics and Automation.

[18] Lee D., Lim H., Han S. (2025). GenZ-ICP: Generalizable and Degeneracy-Robust LiDAR Odometry Using an Adaptive Weighting. IEEE Robot. Autom. Lett..

[19] Li C., Pan W., Yuan X., Huang W., Yuan C., Wang Q., Wang F. (2024). High-Precision Map Construction in Degraded Long Tunnel Environments of Urban Subways. Remote Sens..

[20] Xin R., Guo N., Ma X., Liu G., Feng Z. (2024). RLI-SLAM: Fast Robust Ranging-LiDAR-Inertial Tightly-Coupled Localization and Mapping. Sensors.

[21] Nissov M., Khedekar N., Alexis K. Degradation Resilient LiDAR-Radar-Inertial Odometry. Proceedings of the IEEE International Conference on Robotics and Automation.

[22] Filip I., Pyo J., Lee M., Joe H. (2023). LiDAR SLAM with a Wheel Encoder in a Featureless Tunnel Environment. Electronics.

[23] Kurz G., Scherer S.A. When Geometry Is Not Enough: Using Reflector Markers in Lidar SLAM. Proceedings of the IEEE/RSJ International Conference on Intelligent Robots and Systems.

[24] Wang S., Chen X., Ding G., Li Y., Xu W., Zhao Q., Gong Y., Song Q. (2021). A Lightweight Localization Strategy for LiDAR-Guided Autonomous Robots with Artificial Landmarks. Sensors.

[25] Zeng Q., Kan Y., Tao X., Hu Y. (2021). LiDAR Positioning Algorithm Based on ICP and Artificial Landmarks Assistance. Sensors.

[26] Huang J.K., Wang S., Ghaffari M., Grizzle J.W. (2021). LiDARTag: A Real-Time Fiducial Tag System for Point Clouds. IEEE Robot. Autom. Lett..

[27] Liu Y., Schofield H., Shan J. (2022). Intensity Image-Based LiDAR Fiducial Marker System. IEEE Robot. Autom. Lett..

[28] Liu T., Chang L., Niu X., Liu J. (2020). Pole-Like Object Extraction and Pole-Aided GNSS/IMU/LiDAR-SLAM System in Urban Area. Sensors.

[29] Schaefer A., Büscher D., Vertens J., Luft L., Burgard W. (2021). Long-Term Vehicle Localization in Urban Environments Based on Pole Landmarks Extracted from 3-D Lidar Scans. Robot. Auton. Syst..

[30] Dong H., Chen X., Särkkä S., Stachniss C. (2023). Online Pole Segmentation on Range Images for Long-Term LiDAR Localization in Urban Environments. Robot. Auton. Syst..

[31] Besl P.J., McKay N.D. (1992). A Method for Registration of 3-D Shapes. IEEE Trans. Pattern Anal. Mach. Intell..

[32] Pomerleau F., Colas F., Siegwart R., Magnenat S. (2013). Comparing ICP Variants on Real-World Data Sets. Auton. Robot..

[33] Kaess M., Johannsson H., Roberts R., Ila V., Leonard J.J., Dellaert F. (2012). ISAM2: Incremental Smoothing and Mapping Using the Bayes Tree. Int. J. Robot. Res..

[34] Xu Z., Guo S., Song T., Zeng L. (2020). Robust Localization of the Mobile Robot Driven by Lidar Measurement and Matching for Ongoing Scene. Appl. Sci..

[35] Kashani A.G., Olsen M.J., Parrish C.E., Wilson N. (2015). A Review of LIDAR Radiometric Processing: From Ad Hoc Intensity Correction to Rigorous Radiometric Calibration. Sensors.

[36] Kaasalainen S., Jaakkola A., Kaasalainen M., Krooks A., Kukko A. (2011). Analysis of Incidence Angle and Distance Effects on Terrestrial Laser Scanner Intensity: Search for Correction Methods. Remote Sens..

[37] Tan K., Chen J., Qian W., Zhang W., Shen F., Cheng X. (2019). Intensity Data Correction for Long-Range Terrestrial Laser Scanners: A Case Study of Target Differentiation in an Intertidal Zone. Remote Sens..

[38] Liu G., Gao W., Pan S. (2025). Analysis of Factors Affecting Random Measurement Error in LiDAR Point Cloud Feature Matching Positioning. Remote Sens..

[39] Vinodkumar P.K., Karabulut D., Avots E., Ozcinar C., Anbarjafari G. (2023). A Survey on Deep Learning Based Segmentation, Detection and Classification for 3D Point Clouds. Entropy.

[40] Alaba S.Y., Ball J.E. (2022). A Survey on Deep-Learning-Based LiDAR 3D Object Detection for Autonomous Driving. Sensors.

[41] Singh D.P., Yadav M. (2024). Deep Learning-Based Semantic Segmentation of Three-Dimensional Point Cloud: A Comprehensive Review. Int. J. Remote Sens..

[42] Luo W., Xing J., Milan A., Zhang X., Liu W., Kim T.K. (2021). Multiple Object Tracking: A Literature Review. Artif. Intell..

[43] Bewley A., Ge Z., Ott L., Ramos F., Upcroft B. Simple Online and Realtime Tracking. Proceedings of the IEEE International Conference on Image Processing.

[44] Kuhn H.W. (1955). The Hungarian Method for the Assignment Problem. Nav. Res. Logist. Q..

[45] Ester M., Kriegel H.P., Sander J., Xu X. A Density-Based Algorithm for Discovering Clusters in Large Spatial Databases with Noise. Proceedings of the Second International Conference on Knowledge Discovery and Data Mining.

